# Strategies for engineering biomimetic lipoproteins to improve drug delivery efficiency and tumor therapy

**DOI:** 10.1007/s13346-026-02160-3

**Published:** 2026-05-29

**Authors:** Yuchen He, Naibo Yin, Zhiwen Zhang, Guo-Liang Lu, Jingyuan Wen

**Affiliations:** 1 School of Pharmacy, Faculty of Medical and Health Sciences, the University of Auckland, Auckland, 1023 New Zealand; 2 Auckland Cancer Society Research Centre, Faculty of Medical and Health Sciences, the University of Auckland, Auckland, 1023 New Zealand; 3 School of Pharmacy, Fudan University, Shanghai, China; 4 Maurice Wilkins Centre, the University of Auckland, Auckland, 1023 New Zealand

**Keywords:** Lipoprotein, Drug delivery, Cancer treatment, Chemotherapy, Immunotherapy, Nucleic acids drugs

## Abstract

Cancer treatment remains limited by key challenges, including poor drug biodistribution, restricted tumor penetration, and severe systemic toxicity. Lipoprotein nanoparticles (LPNs) have emerged as promising platforms for drug delivery, addressing these obstacles. Their inherent biocompatibility, small size, and natural ability to interact with specific cellular receptors make them ideal candidates for targeted cancer therapy. This review presents recent advances in the design of LPNs to improve the precision and efficiency of anti-tumor drug delivery. We investigate how these smart nanoparticles enhance the therapeutic performance of three primary treatment modalities: chemotherapy, immunotherapy, and emerging nucleic acid-based therapies. This comprehensive review summarizes current strategies in the field and offers forward-looking insights into future innovations in LPN drug delivery systems (DDSs) for cancer therapy.

## Introduction

Cancer is a significant global health issue, with around 20 million new cases and 9.7 million deaths [1]. Numerous effective anti-cancer drugs have been developed over the past decades. However, cancer treatment continues to face significant challenges, such as systemic toxicity, multidrug resistance, limited tumor targeting, poor penetration into tumor sites, inadequate drug stability, and low aqueous solubility [2]. Therefore, optimizing therapeutic efficacy and enhancing bioavailability remain significant challenges for new and already approved anticancer agents. Drug delivery systems (DDSs) are specifically engineered to improve the pharmacokinetic and pharmacodynamic profiles of therapeutic compounds, thereby enhancing their clinical performance and reducing side effects. Nanoparticles are a type of DDSs that offer intrinsic advantages over conventional drug carriers. With sizes typically ranging from 1 to 1000 nm and a high surface-area-to-volume ratio, they enable prolonged circulation and enhanced drug delivery. Their structural versatility allows for improved solubility, protection of therapeutic agents, and controlled or sustained release.

Furthermore, the surface modifications of DDSs can enable targeted delivery, reducing systemic toxicity and improving efficacy. Some nanoparticles can cross biological barriers and respond to stimuli such as pH or enzymes. These features make nanoparticles highly promising for advancing treatment outcomes, especially in cancer therapy [3–6]. Based on fabrication methods and materials, nanoparticles can be broadly classified into polymeric nanoparticles, lipid nanoparticles, and inorganic nanoparticles [3]. Among these, lipid nanoparticles have drawn a lot of attention due to their unique features, including biocompatibility, biodegradability, enhanced drug solubility, protection against drug degradation, and the ability to facilitate targeted delivery and controlled release. These properties make lipid nanoparticles particularly valuable in pharmaceutical and biomedical applications.

Lipoprotein nanoparticles (LPNs) are a type of lipid nanoparticle composed of lipids and proteins that have emerged as highly promising candidates for drug delivery systems. Lipoproteins, originally as endogenous carriers, are produced in the liver and intestines and can transport cholesterol and triglycerides between the liver and the other organs [7]. Natural LPNs can be isolated from various biological fluids, including blood, cerebrospinal fluid, lipoaspirate, and conditioned media from hepatocyte cell lines [8]. However, the isolation methods of natural lipoproteins are generally time-consuming, less scalable, difficult to standardize, and always result in co-separation with other components [9]. Therefore, reconstituted LPNs and synthetic LPNs have emerged as alternatives. Notably, these two terms are sometimes used interchangeably in the literature, leading to confusion. In a technical sense, reconstituted LPNs typically refer to particles assembled from purified full-length apolipoproteins, either naturally isolated or produced recombinantly. In contrast, synthetic LPNs typically refer to nanoparticles composed of short apolipoprotein-mimetic peptides generated via chemical synthesis (e.g., solid-phase peptide synthesis) and lipids, mimicking the structure and function of native lipoproteins [8, 10–13]. Both reconstituted LPNs and synthetic LPNs are analogous to endogenous lipoproteins and retain the biocompatibility, stability, and immunoevasion properties, thereby preventing them from eliciting an immunological response [7, 8].

The endogenous origin and physiological role provide LPNs with circulatory stability and a favorable safety profile. Reconstituted and synthetic LPNs, by highly mimicking the structure of natural LPNs, have been reported to exhibit comparable biocompatibility, stability, targeting ability, and immunoevasion capability [8]. In particular, reconstituted LPNs exhibit no detectable cytotoxicity in human hepatic carcinoma cells at concentrations up to 320 µg/mL and do not induce acute toxicity or measurable antibody responses in the murine model. In the absence of a cargo molecule, reconstituted LPNs also elicit no immunogenic response [14]. In comparison, safety data for synthetic LPNs are mostly available only from individual studies, which indicate that they are safe and biocompatible for human use [15]. The stability of LPNs can be influenced by their lipid composition individually [14] and their shape [16, 17]. With different lipids, the maximum t₁/₂ of LPNs can vary from 128.8 h (1, 2-dioleoyl-sn-glycero-3-phosphocholine, DOPC) to only 8.3 h (1, 2-dimyristoyl-sn-glycero-3-phosphocholine, DMPC) [14]. Discoidal HDLs undergo in vivo remodeling induced by lecithin-cholesterol acyltransferase (LCAT), which can trigger cargo leakage; this has prompted notable modifications to the nanodiscs design [16].

**Fig. 1:**
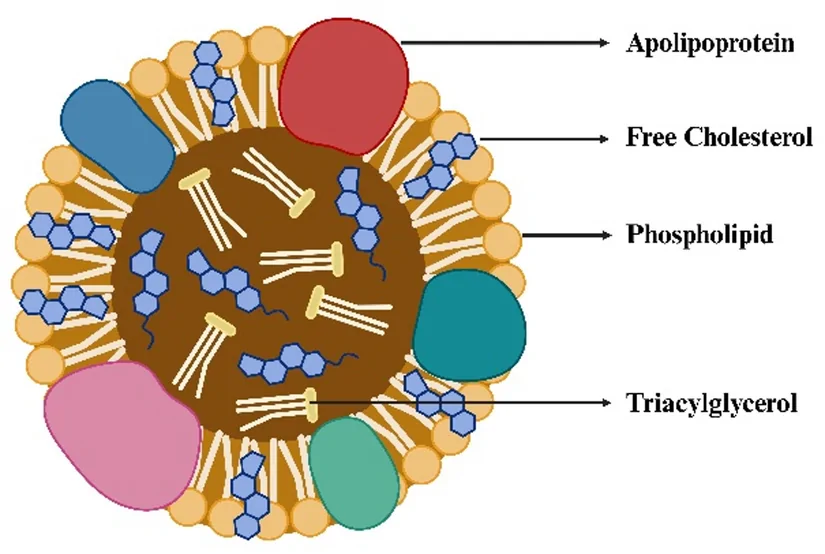
Structure of lipoprotein nanoparticles (LPNs). The core is rich in hydrophobic triacylglycerol and cholesterol ester, and the outer shell is formed by a monolayer of apolipoprotein with amphiphilic groups, phospholipids, and free cholesterol [18]

LPNs are typically spherical particles, although they can also be disc-shaped. The core is rich in hydrophobic triacylglycerol and cholesterol ester, and the outer shell is formed by a monolayer of apolipoprotein (apo) with amphiphilic groups, phospholipids, and free cholesterol (Fig. 1) [18]. Based on size, density, and lipid-to-protein ratios, lipoproteins can be classified into five major categories, including chylomicron (CM), very low-density lipoprotein (VLDL), intermediate-density lipoprotein (IDL), low-density lipoprotein (LDL), and high-density lipoprotein (HDL) (Table 1). The particle sizes of CM (~ 80–1000 nm) and VLDL (~ 30–80 nm) are much larger, which restricts their deep penetration into tumor tissue, as the spacing between collagen fibrils in the tumor extracellular matrix (ECM) is typically less than 40 nm [19], preventing them from being effective tumor drug carriers. IDL, a short-lived intermediate in the VLDL-to-LDL conversion pathway, is rapidly cleared by the liver or further processed into LDL after partial lipolysis of VLDL. This brief residence time means it cannot provide a stable or predictable circulation window for drug delivery [8]. Therefore, HDL and LDL, with sizes commonly under 30 nm, can penetrate deeply into the tumor site and exhibit extended circulation time in the human body [7], and are exceptionally well-suited as drug carriers. In addition, LDL is enriched in apoB-100 and specifically binds to the LDL receptor (LDLR), which is highly upregulated in rapidly proliferating tumor cells due to their increased demand for cholesterol for new membrane synthesis, thereby enabling efficient receptor-mediated endocytosis and lysosomal release of encapsulated drugs [20]. LDL (~ 20 nm) is slightly larger than HDL, potentially allowing it to carry a larger drug payload [11]. HDL, with its ultra-small size (~ 5–10 nm), can potentially penetrate deeper into tumor tissue. HDL mainly binds to scavenger receptor class B type 1 (SR-B1) via ароA-1. SR-B1 is also abundantly expressed in many tumors due to the high demand for cholesterol/cholesteryl esters for new membrane synthesis and steroidogenesis within tumors. However, unlike LDL, SR-B1-mediated tumor targeting does not internalize the entire particle. Instead, it forms a hydrophobic channel across the plasma membrane, allowing cholesteryl esters and other cargo incorporated into the HDL core to move directly into the cytosol. Because the particle itself remains at the membrane, the cargo transfer is non-endocytic. Thus, drugs carried by HDL are delivered without being trafficked to endosomes or lysosomes, thereby avoiding the acidic, degradative environment, which is suitable for protecting fragile payloads such as siRNA, antisense oligonucleotides, or labile peptides [7, 8, 20, 21]. All these characteristics make LDL and HDL promising drug nanocarriers for cancer treatment.

LPNs, especially LDL and HDL, offer multiple advantages as drug carriers, including biocompatibility, good stability, non-immunogenicity, natural targeting to tumor sites, ease of modification, and versatility for carrying various therapeutic agents [8, 22]. All these properties make LPNs versatile drug carriers, especially in anti-tumor treatment. This review highlights design strategies for using LPNs as drug-delivery systems to enhance delivery efficiency and improve tumor treatment outcomes. It discusses methods for loading drugs into LPNs and how LPNs address the challenges associated with delivering chemotherapeutic agents, immunotherapeutics, and nucleic acids to tumor sites, while exploring the design principles of LPNs tailored to different classes of anticancer drugs.

A comprehensive literature search was conducted using the Web of Science Core Collection to identify relevant publications concerning the application of LPNs as drug delivery systems for tumor therapy. The search was performed using the following combination of topic search (TS) terms: (“lipoprotein” AND “tumor” AND “drug delivery”). The search was restricted to articles published between 2019 and 2025 to ensure the inclusion of contemporary advances in the field. Only peer-reviewed original research articles and review articles published in English were considered. Studies were included if they met the following criteria: (i) lipoproteins were employed as drug delivery vehicles; and (ii) the investigation focused on tumor treatment. Studies were excluded if lipoproteins were discussed solely as biomarkers or endogenous disease-related molecules without a drug delivery function, or if they were not relevant to tumor treatment. Priority was given to articles published in peer-reviewed journals of recognized standing within the fields of drug delivery, nanomedicine, and pharmaceutical sciences.

**Table 1:** Physical characteristics of the five major lipoprotein categories [8]

Lipoprotein	Density (g/cm3)	Size (nm)	Major apolipoprotein	Origin	Primary function	Major lipids
Chylomicrons	< 0.950	80-1000	aроB-48, ароС-II, ароA-1, ароА-II, apoA-IV, apoE	Intestines	Transport of dietary triglycerides to non-hepatic tissues	Triglycerides
VLDL	0.930–1.006	30–80	aрoB-100, ароС, apoE	Liver, intestines	Transport of endogenous triglycerides to non-hepatic tissues	Triglycerides
IDL	1.006–1.019	~ 25	арoB-100, ароС, apoE	Catabolism of VLDL	Intermediate of VLDL and LDL	Cholesterol
LDL	1.019–1.063	~ 20	apoB-100	Catabolism of IDL	Transport of most of the cholesterol to non-hepatic tissues	Cholesterol
HDL	1.063–1.210	5–10	ароA-1, ароА-II, ароС, ароE	Liver, intestines, other	Transport of cholesterol from non-hepatic tissues to the liver	Cholesterol, phospholipids

## Methods of loading anti-tumor drugs to LPNs

Depending on the physicochemical properties of the agent being incorporated, the loading method can be categorized into three main strategies for LPNs: surface loading, covalent modification, and core loading via reconstitution. Surface loading utilizes non-covalent intercalation, in which amphiphilic molecules partially embed in the LPNs’ surface while exposing hydrophilic moieties to the aqueous phase. Loading efficiency and stability depend on the balance of intermolecular forces [23]. The covalent modification method involves conjugating therapeutic agents to the apolipoprotein surface or to the phospholipid headgroup [19]. Core loading via reconstitution encapsulates hydrophobic molecules in the nanoparticle core, where hydrophobic agents are entrapped through lipid exchange during lyophilization and organic extraction. Table 2 compares these loading methodologies.

**Table 2:** Drug loading methods of lipoprotein

Loading strategy	Method	Mechanism	Suitable drug types	Advantages	Disadvantages	Ref
Surface loading	Non-covalent intercalation	Amphiphilic agents are embedded into the phospholipid shell via van der Waals forces.	Amphiphilic drugs	Simple, no chemical modification required; suitable for amphiphilic drugs.	Low binding stability; potential drug leakage due to weak interactions.	[15, 23–30]
Covalent modification	Protein/phospholipid conjugation	Drugs or targeting ligands are covalently attached to surface residues (e.g., lysine, cysteine).	Targeting ligands; Imaging agents	High stability; precise control over drug loading; enables active targeting.	May alter natural lipoprotein structure; requires optimization.	[10, 31–46]
Core loading	Reconstitution	Hydrophobic core lipids are replaced with drugs via lyophilization or solvent extraction.	Hydrophobic drugs	High payload capacity; ideal for hydrophobic drugs (e.g., chemotherapeutics).	Complex process; potential structural disruption.	[10, 11, 15, 16, 24, 25, 28– 30, 32– 34, 36, 38, 40, 42, 44, 46– 55]
	Peptide-phospholipid scaffold (HPPS)	Synthetic peptides (e.g., 18 A) self-assemble with phospholipids to form HDL-like nanoparticles.	Cytosolic-acting agents, such as siRNA	Avoids endosomal degradation of natural protein	Requires validation of biofunctionality; scalability challenges.	[56, 57]
	Inorganic Core Hybrids	Lipoproteins encapsulate inorganic cores	Imaging agents: • Quantum dots • Gold nanoparticles	Enables multimodal imaging (CT, fluorescence); enhanced stability.	Potential biocompatibility concerns; complex synthesis.	[20, 58, 59]

LPNs exhibit distinctive advantages as drug carriers through various drug-loading methods according to drug type and research purpose. For example, core reconstruction (mainly facilitating the encapsulation of hydrophobic chemotherapeutic agents) and surface covalent modification (enabling the conjugation of targeting ligands) address the requirements for significant drug loading capacity and precise targeting, respectively. Currently, researchers increasingly implement multimodal loading strategies combining core drug encapsulation with surface modification by targeting agents to achieve substantial drug loading capacity and targeted delivery simultaneously. Researchers also explore using covalent methods first to combine different drugs, then loading them into the core or incorporating them onto the surface. This ability to incorporate multiple drugs or modification agents simultaneously via distinct strategies makes LPNs versatile carriers capable of fulfilling multiple therapeutic purposes.

## Deliver chemotherapeutic drugs by LPNs

Despite chemotherapy being the cornerstone of systemic cancer treatment, conventional chemotherapeutic drugs still face several significant challenges that limit their therapeutic efficacy [8]. The primary challenges include poor drug solubility, biological barriers hindering drug uptake, lack of tumor specificity, and the development of multidrug resistance (MDR). LPNs serve as a promising drug-delivery platform to address these limitations, combining their endogenous biocompatibility, tumor-targeting, ease of modification, and multidrug-loading capacity.

### Improving the solubility and stability of hydrophobic chemotherapeutics

Many classic chemotherapeutic agents are highly hydrophobic, leading to poor solubility in physiological fluids and reduced bioavailability [2]. These often necessitate the use of toxic organic solvents or surfactants as solubilizers, which can cause severe side effects [2, 8]. LPNs have emerged as an excellent nanocarrier to address this issue. Their hydrophobic lipid core, stabilized by amphipathic apolipoproteins and a phospholipid monolayer, intrinsically enables them to encapsulate hydrophobic drugs, such as Taxane [16, 29, 60], Anthracyclines [34, 49], natural products [55], and gas messengers [48], et al., within their lipid cores while maintaining water solubility through their hydrophilic shell [2].

**Fig. 2:**
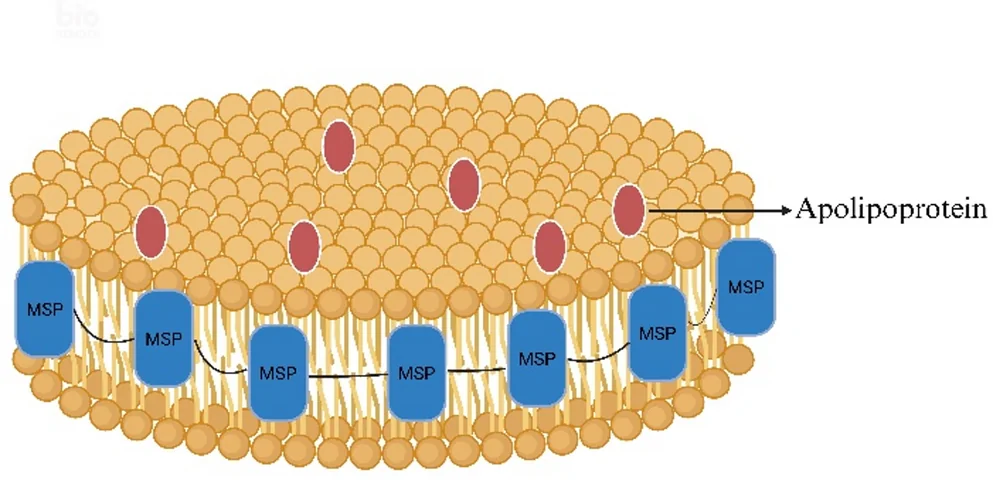
Stabilize the nanodiscs by wrapping the lipid bilayers with membrane scaffold proteins (MSP). MSP is an amphipathic protein derived from human apoA1, and its helices wrap around the lipid bilayer like a belt [29]

Although LPNs are naturally advantageous for delivering hydrophobic drugs, advanced modifications can further improve their performance. Nanodiscs are disc-shaped LPNs reported to be unstable in plasma [17]. An allosteric reaction catalyzed by serum LCAT causes cholesterol and unsaturated fatty acids of lecithin in HDL to generate cholesterol esters (CE) and lysophospholipids, resulting in drug leakage from disc-shaped HDL and reducing its targeting efficiency [16]. To address this issue, Pandey et al. [29] stabilized the nanodiscs by wrapping lipid bilayers 1-palmitoyl-2-oleoyl-glycero3-phosphocholine (POPC) with membrane scaffold proteins (MSPs). MSP is an amphipathic protein derived from human apoA1, and its helices wrap around the lipid bilayer like a belt (Fig. 2). This results in an initial burst release of 35% of the drug and sustained release over 24 h. The drug loading capacity correlated positively with the hydrophobicity (Log P) of the drugs tested. This MSP-wrapped nanodisc loaded with Cabazitaxel got 20% higher cytotoxicity than free Cabazitaxel in 3D cell models. Li et al. [16] also developed a Paclitaxel-loaded discoid-reconstituted HDL (d-rHDL), which can also avoid LCAT-catalyzed allosteric reaction. Unlike Pandey, they substituted the original cholesterol in d-rHDL with a modified cholesterol, n-butyraldehyde bi-cholesterol (BCC). The 3’-hydroxyl group of BCC is protected by an acetal bond, which protects BCC from LCAT-induced esterification. Docetaxel (DTX) can also be loaded into oleic acid (OA)-modified mesoporous silica nanoparticles (OA-MSNs) conjugated with bovine α-lactalbumin (MSN-BAMLET), a lipoprotein complex [60]. The tailored mesostructure of MSN makes it a high-surface-area, large-pore-volume, and non-toxic drug delivery system. OA provides a hydrophobic surface for loading DTX via hydrogen bonds and serves as an anchor point for binding α-lactalbumin. Compared with a non-modified group, the DTX loading efficiency of OA-MSNs increased from 43.5% to 73.2%. However, a slight degradation of the nanoparticles was observed over time, but the complete biodegradation process and its products were not fully characterized. Besides DTX, bovine α-lactalbumin (BLA) is also a well-known protein-based anticancer agent, but exhibits strong hemolytic activity, and its activity can be weakened by serum. The biocompatibility issues of BLA limit its application in endovascular treatment. However, when BLA is anchored to the surface of OA-MSNs through a hydrothermal approach, the immobilization appears to constrain the BLA’s interaction with red blood cells. The hemolysis assay shows that MSN-BAMLET exhibits strong hemocompatibility up to 400 µg/ml, a significant improvement over BLA alone. Kumari et al. [53] designed Curcumin (CUR)-loaded human serum albumin (HSA) based LPNs conjugated with dioleoyl phosphoethanolamine (CUR-HSA-DOPE NPs) for breast cancer therapy and achieved significantly improved tumor inhibition compared with the free CUR. They took advantage of HSA’s natural properties, including excellent biocompatibility, high stability, strong drug-binding capacity, and a long biological half-life (approximately 19 days). HSA is also highly soluble (up to 40% w/v at physiological pH), resistant to temperatures up to 60 °C, and it possesses inherent tumor-targeting abilities via SPARC and GP60 receptors, which are overexpressed in many cancer cells.

### Synergistic therapy

Co-delivering multiple therapeutic modalities through LPNs can achieve enhanced health outcomes greater than the sum of their individual benefits.

Abnormal tumor vasculature impedes effective drug delivery, but innovative combination therapy delivered by LPNs offers promising strategies to either normalize blood vessels or strategically destroy them, thereby significantly enhancing therapeutic efficacy in cancer treatment. Blood vessels surrounding tumors grow in a dysregulated manner due to the excessive production of proangiogenic factors, such as vascular endothelial growth factor (VEGF) [48]. These abnormal vessels are characterized by structural tortuosity, inadequate pericyte coverage, improper perfusion, and high permeability. The disorganized structure creates several problems. It leads to elevated interstitial fluid pressure and impaired blood flow, which ultimately hampers drug delivery deep into the tumor and creates areas of hypoxia [10, 48]. Wu et al. [48] developed a two-step treatment strategy using NO-BLP (Nitric oxide donor-loaded bioinspired lipoprotein) to normalize tumor vessels, followed by the administration of albumin-bound Paclitaxel nanoparticles (PAN). NO is a downstream signal molecule of phosphoinositide 3-kinase-Akt-endothelial nitric oxide synthase (PI3K-Akt-eNOS) cascade signaling pathway [61], which can be activated by VEGF. The increasing NO generation activates the soluble guanylyl cyclase-cyclic GMP-protein kinase G (sGC–cGMP–PKG) signaling axis in endothelial cells and perivascular smooth muscle, which shifts tumor vasculature from abnormal, leaky, and hypoperfused to normalized, stable, and adequately perfused, and thus collectively facilitates deeper intratumor penetration and higher accumulation for the following or combining therapeutic agents [62]. The issues of a very short in vivo half-life for NO and poor tumor accumulation due to rapid diffusion away from the site of action can be solved by LPNs. The results showed that NO-BLP treatment achieved significantly higher pericyte coverage, improved vessel perfusion, and almost absent hypoxia signals, indicating a more mature, stable, and functional vessel wall. The sequential treatment with NO-BLP and PAN also resulted in an 81.03% tumor growth inhibition rate, which was significantly higher than that with PAN monotherapy (52.41%). Besides normalizing vessels around tumors, directly cutting off the tumor’s blood supply can also be a smart way to promote tumor death synergistically. Xu et al. [10] achieved this goal by co-delivering photoactivatable tumor-infarction therapy and chemotherapy via synthetic HDL nanodiscs. The nanodiscs are modified with a neo-vasculature-targeting peptide (RGD), thereby enabling them to bind to tumor vessel endothelial cells. Under laser irradiation, a photodynamic drug, Pyropheophorbide-a (Ppa), is activated, generating reactive oxygen species (ROS) to damage tumor vasculature endothelial cells. This photodynamic vascular damage can potentially activate the natural coagulation cascade, leading to local thrombosis formation within tumor vessels and cutting off the tumor’s nutrient supply. At the same time, a chemotherapy drug, Camptothecin (CPT), will be released in the tumor microenvironment to kill cancer cells synergistically.

Unlike the typical injection administration for LPNs, Yang et al. [11] tried to develop orally administered reconstituted apolipoprotein B lipoparticles (rABLs) to simultaneously and locally deliver two epigenetic drugs, demethylating agent 5-aza-2’-deoxycytidine (5AZA) and histone deacetylase (HDAC) inhibitors trichostatin A (TSA) or LBH589, for the treatment of gastric cancer. Both demethylating agents and HDAC inhibitors can reactivate tumor suppressor genes, arrest the cell cycle, and promote apoptosis. Due to the acidic environment in the stomach, this study tested the drugs released from the rABLs at pH 1.5, 3.5, and 5.5, with pH 1.5 (similar to stomach acidity) showing the highest release rate. However, the authors noted that the drug-loaded rABLs did not exhibit better antitumor effects than the free drugs in their mouse model, possibly due to the higher gastric pH in mice (pH 3.1 to 4.5) compared to humans (pH 1.5 to 3.5), which led to reduced drug release in the mouse model.

### Tumor targeting of LPNs

The non-specific distribution of chemotherapeutic drugs throughout the body results in inadequate concentration at tumor sites while causing significant toxicity to healthy tissues, including cardiotoxicity, myelosuppression, and damage to other organs, which frequently necessitates dose reduction or discontinuation of treatment [8]. This poor targeting efficiency means only about 0.01–0.1% of administered doses of small-molecule drugs can reach tumor tissues [63, 64]. Additionally, various biological barriers, including dense ECM around the tumor, the blood-brain barrier, enzymatic degradation, and rapid clearance by the reticuloendothelial system (RES), significantly impede drug delivery to tumor sites [65].

LPNs represent a promising strategy to overcome the poor targeting efficiency of conventional chemotherapeutics, as they naturally selectively deliver various cargos to cancer cells via the overexpression of LDLR and SR-B1 on the surface of multiple carcinoma cells [57]. The mechanisms of tumor targeting for LPNs can involve two aspects: passive tumor uptake and active targeting. (i) Passive tumor uptake: a non-specific tumor accumulation of nanoparticles in tumors through the enhanced permeability and retention (EPR) effect. Traditionally, this means that ultra-small nanoparticles can extravasate from the abnormally leaky tumor vessels and remain at the tumor site [66, 67]. In addition, LPNs are inherently biocompatible, and their endogenous identity reduces the risk of being uptaken by the RES, thereby further prolonging their plasma residence time and enhancing tumor exposure [19]. (ii) Active targeting: including native receptor-mediated active tumor targeting and enhanced tumor targeting by targeting modification.

#### Native receptor-mediated active tumor targeting

LDL binds specifically to the LDLR via apoB-100, leading to clathrin-mediated endocytosis into endo/lysosomes, where the core cargo is released. Many malignancies upregulate LDLR and display tumor-selective LDL uptake, enabling a tumor-specific drug delivery of LDL [20]. After being loaded into LDL, chemotherapeutics such as Doxorubicin can achieve significantly higher drug uptake in LDLR-overexpressing cancer cells than in cells with relatively low LDLR expression, thereby improving therapeutic outcomes and reducing side effects [24]. HDL binds specifically to SR-B1 through apoA-I mainly, but as Smith et al. reported, apoA-II exhibits a stronger binding affinity to SR-B1 than apoA-I, potentially indicating that the targeting efficiency of HDL might be further modulated through apolipoprotein selection [57]. SR-B1 is also frequently overexpressed on many tumors. However, unlike LDLR-mediated endocytosis, SR-B1–mediated HDL uptake is non-endocytic. It means that after binding to SR-B1, it forms a hydrophobic channel across the plasma membrane, enabling the direct uptake of core-lipid and core-loaded cargo into the cytoplasm, largely bypassing endo/lysosomal trapping, which makes HDL more attractive as an ideal carrier for fragile cargos that are sensitive to being damaged by endo/lysosome, such as siRNA, plasmids, and photosensitizers [2, 35, 57–69]. Ouyang et al. [70] incorporated the p53 plasmid into HDL. The results showed that the HDL delivered cargo into the cytoplasm without obvious colocalization with endosomal signals, whereas the non-HDL control mainly colocalized with endosomes, indicating endocytic uptake. However, recent studies tend to interpret the SR-B1-mediated endo/lysosomal escaping mechanism more cautiously. Kachan et al. [71] stated that although a predominantly cytosolic distribution and limited lysosomal co-localization of siRNA were observed in HepG2 cells, the same siRNA-loaded HDL showed significant siRNA and lysosome co-localization in THP-1-derived macrophages, indicating the SR-BI-mediated endo/lysosomal escape of HDL might be cell-type-dependent.

Some studies indicated that SR-B1-mediated direct cytoplasmic drug delivery may have the potential to overcome the multidrug resistance (MDR) [2, 69, 72]. MDR is a specific form of drug resistance in which cancer cells become resistant to multiple structurally and functionally unrelated anticancer agents. MDR is associated with a variety of mechanisms, including enhanced drug efflux, genetic factors, growth factors, and increased xenobiotic metabolism. Among them, P-glycoprotein (P-gp)–mediated drug efflux is a well-established MDR mechanism in a variety of tumors. A significant correlation between increased P-gp expression and enhanced chemotherapeutic resistance has been reported. P-gp, present in the cell membrane, pumps chemotherapeutic agents out of cells, reducing intracellular drug accumulation when these agents are exposed to the membrane [69, 72, 73]. Therefore, direct cytoplasmic drug delivery without exposing the drug to the membrane appears to bypass the membrane pumps that confer drug resistance [69, 72]. Although the idea is attractive, there is little direct evidence to support it so far. Zhang et al. used reconstituted HDL to co-deliver Paclitaxel and the P-gp-mediated MDR reversal agent HZ08, achieving prolonged intracellular Paclitaxel retention and enhanced apoptosis induction in PTX-resistant MCF-7 human breast cancer cells [74], which attributes the MDR reversal effect to HZ08, rather than to SR-B1-mediated cytoplasmic drug delivery. Li et al. co-delivered an antisense oligonucleotide (antimiRNA21) and Sorafenib. By knocking down miR-21, they observed reduced expression of hypoxia-inducible factor-1α (HIF-1α), which has been reported to be involved in Sorafenib resistance in hepatocellular carcinoma (HCC). However, the improved resistance-related outcomes were mainly attributed to the nucleic acid agent, not to HDL’s cytoplasmic payload release. Moreover, the results are still limited, as they all came from regular HepG2 cells rather than a Sorafenib-resistant model [75]. Therefore, the evidence remains insufficient to support the notion that SR-B1-mediated direct cytoplasmic drug delivery contributes to overcoming MDR, and existing studies still focus more on general chemotherapeutic resistance than on MDR specifically.

In addition to targeting cancer cells that form the bulk of the tumor mass, LPNs can also naturally target a small subset of cancer stem cells (CSCs) present in tumor tissues through native receptor-mediated way [67]. On the surface of CSCs, high levels of LDL receptor-related protein 1 (LRP-1) are expressed and can be recognized by LPNs. LRP and LDLR are both members of the LDLR family of endocytic receptors. LRP recognizes a wide spectrum of structurally and functionally unrelated ligands, including coagulation factor VIII (FVIII), while the ligand of LDLR is restricted to apoB-100 and apoE. Stem cells are the longest-living and self-renewing cells in many tissues and are therefore highly likely to harbor the initial oncogenic mutation. CSCs can survive toxins and genotoxic stress through different mechanisms, such as upregulating DNA repair pathways, inducing expression of antiapoptotic genes, and maintaining low levels of ROS. Thus, CSCs are partially resistant to standard chemotherapy and radiotherapy [76]. It has been reported that tumors exploit the self-renewal capacity of stem cells to sustain long-term proliferation and tissue repair, thereby facilitating invasion [77], suggesting that the frequency of stem cells in tumor tissues is associated with prognosis. Yin et al. [51] incorporated salinomycin (Sal), an effective anticancer drug particularly active against CSCs, into HDL, forming S-HDL, for the treatment of cervical cancer. The results showed higher cellular uptake of S-HDL by both cervical cancer cells and cervical cancer stem cells (CCSCs). The S-HDL also effectively prevented CCSCs from forming tumor spheres, compared with free salinomycin, indicating that the self-renewal characteristics of CCSCs were inhibited.

#### Enhanced tumor targeting by targeting modification

Although LPNs exhibit a natural targeting ability to tumor cells, the SR-B1 and LDLR families are also expressed in normal tissues, suggesting that relying solely on these receptors may not achieve high tumor specificity, especially when another biological barrier is present. Therefore, researchers began conjugating or decorating LPNs with unique targeting agents that bind to other specific receptors on tumor cell surfaces, thereby providing superior tumor targeting and penetration.

Because natural apolipoproteins are difficult and expensive to isolate from human serum and may pose potential biosafety concerns, serum albumin (BSA) has become a more accessible alternative to apolipoproteins for synthesizing LPNs [45]. Beyond increasing the stability of nanoparticles and helping to avoid recognition by the RES, BSA can also serve as a targeting agent of HDL nanoparticles by binding to the glycoprotein 60 (GP60) and secreted protein acidic and rich in cysteine (SPARC) receptors on the surface of certain cancer cells (such as hepatoma and breast cancer) [16, 55]. Han et al. [45] further modified BSA with folic acid (FA) when they formulated reconstituted lipoproteins. Folate receptor (FR) distribution is limited in normal tissues but is highly expressed in many cancer cell types, enabling more specific tumor targeting. The dual targeting mechanism resulted in a 1.41-fold increase in drug uptake in cancer cells compared with BSA alone.

**Fig. 3:**
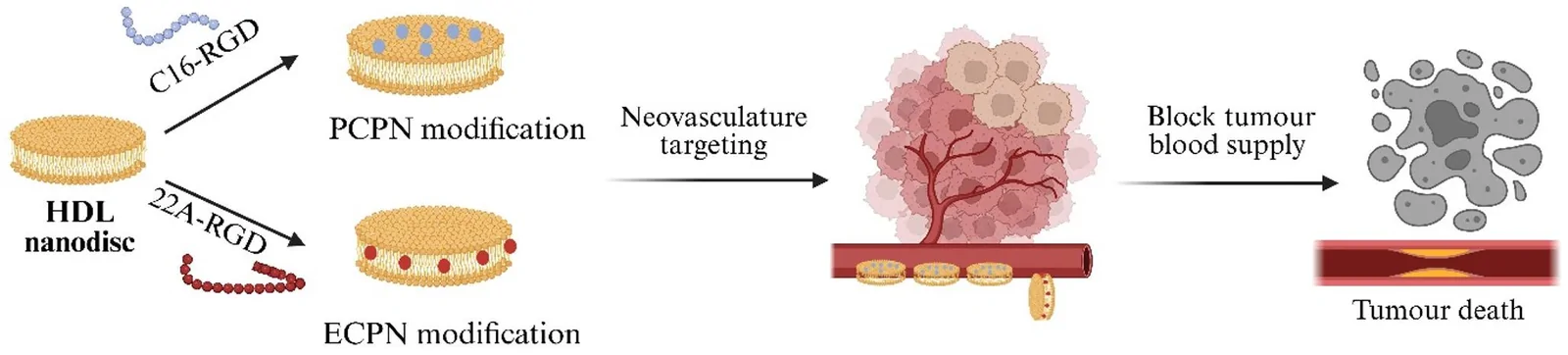
Manipulation of vasculature-targeting capability by neovasculature-targeting peptide (RGD) modification in two different ways. RGD was attached to the planar surfaces (PCPN), meaning the RGD peptide was conjugated to a hydrophobic alkyl chain to create C16-RGD, which was then incorporated into the nanodisc. RGD was attached to the edges (ECPN), indicating that the RGD peptide was linked to the C-terminal end of the apolipoprotein A1 mimetic peptide 22 A, which was then inserted around the edges of the nanodiscs. The nanodiscs were modified with RGD peptide in two different ways to compare the modified nanodiscs’ cellular uptake [10]

More studies focus on modifying LPNs with targeting agents, predominantly peptide-based ligands, to achieve selective tumor delivery. Jiang et al. [40] modified reconstituted HDL nanoparticles with tLyP-1 peptide (tLyP-1-rHDL-PTX/GANT61 NP) to load GANT61 and paclitaxel to treat metastatic triple-negative breast cancer (TNBC). GANT61 is an effective inhibitor of the sonic hedgehog signaling pathway, is aberrantly activated in TNBC, and regulates several malignant phenotypes related to tumor metastasis, but with poor drug bioavailability. tLyP-1 peptide is a ligand of the neuropilin-1 (NRP-1) receptor, which is highly expressed in cancer cells and tumor vessels [34]. tLyP-1 can also promote extravasation and tissue penetration via NRP-1-dependent C-end rule (CendR) internalization, thereby facilitating the nanoparticles’ exit from blood vessels and entry into tumor tissues more effectively. Combined with the natural SR-B1-mediated targeting of HDL, it achieves a dual-targeting characteristic. 3D tumor spheroids testing results showed that tLyP-1-rHDL-PTX/GANT61 NPs penetrated much deeper (over 41.5 μm) than non-modified nanoparticles (12.7–16.5 μm). In vivo data also demonstrated a significantly higher accumulation in tumor tissues compared to other formulations. Given the high expression of legumain in cancer cells, tumor-associated macrophages (TAMs), and the extracellular milieu in tumor regions, Li et al. [43] integrated a legumain-sensitive melittin conjugate into a bioinspired lipoprotein system to achieve enhanced tumor-specific targeting. When this system encounters legumain in the tumor environment, the legumain-sensitive melittin conjugate is cleaved and restored to active melittin, a membrane-lytic peptide that increases membrane permeability. This process can increase the cellular uptake of nanoparticles to 2.65-fold compared to the control formulation. Wang et al. [39] also designed a legumain-activating melittin as a targeting peptide, and they also found that this activation not only enables enhanced tumor penetration but also activates NK cells, reduces M2-like TAMs, and thus designed an immunotherapy-related lipoprotein-based delivery system. CRGDfK is a peptide that specifically recognizes αvβ3/5 integrins, which are highly expressed in cancer cells and tumor vessels. Based on this, Tan et al. [41] designed a CRGDfK peptide-conjugated mertansine decorated biomimetic lipoprotein (BL-RD), loaded with DiD (photosensitizer). The 3D multicellular spheroids (MCS, 4T1 cells) results showed that BL-RD can distribute throughout MCSs to a depth of more than 20 μm, compared with the control group without peptide (BL-D). In vivo photoacoustic imaging also demonstrated better visualization of the interior tumor regions in BL-RD. Tumor section analysis focusing on the extravasation of BL-D and BL-RD from tumor vasculature indicated that BL-D largely co-localized with CD31, suggesting it primarily remained in/near tumor vasculature, while BL-RD was detected at distant sites away from the tumor vessels with strong intensity, demonstrating effective extravasation and diffusion. Xu et al. [10] employed a neovasculature-targeting peptide (RGD) in two different ways (Fig. 3) to modify a synthetic HDL nanodisc. Firstly, RGD was attached to the planar surfaces (PCPN), which means the RGD peptide was conjugated to a hydrophobic alkyl chain to create C16-RGD, then the C16-RGD was incorporated into the lipid membrane of the nanodisc. Secondly, RGD was attached around the edges (ECPN), which means that the RGD peptide was linked to the C-terminal end of the apolipoprotein A1 mimetic peptide 22 A, followed by the 22 A-RGD conjugate, which was inserted around the edge of the nanodiscs. The results showed that PCPN had significantly higher cellular uptake (up to 7-fold) than ECPN. Based on the targeting mechanism, nanodiscs can bind to tumor vessel endothelial cells and then release photodynamic and chemotherapy agents, killing cancer cells.

In addition to targeting peptides, some studies also utilize polysaccharides as targeting agents. Talaat et al. [26] tried to achieve more specific tumor targeting by modifying the surface of LPNs with hyaluronic acid (HA) or chondroitin sulfate (CS) through layer-by-layer coating. Both HA and CS are natural polyanionic glycosaminoglycans that bind to CD44 receptors and are overexpressed on various tumor cells. However, contrary to the initial hypothesis, they found that uncoated LPNs showed comparable or even superior anti-tumor effects to coated LPNs in some cases. They attribute these results to the masking effect of surface modifications, which may hinder LDL receptors’ recognition of LPNs, potentially reducing their natural targeting ability. CS-coated particles showed better results than HA-coated particles at higher doses, attributed to CS’s multiple uptake mechanisms (both CD44 and clathrin-mediated endocytosis) compared to HA’s reliance solely on CD44-mediated uptake. However, they find that HA and CS increased the physical stability of the nanoparticles due to their biocompatibility and biodegradability.

These LPNs can leverage receptor interactions with overexpressed LDLR, SR-B1, and other specific receptors at targeting sites, enabling precise drug delivery to tumor sites while substantially reducing systemic toxicity and off-target effects. The targeting strategies of LPNs are summarized by targeting site and shown in Table 3.

**Table 3:** Tumor targeting strategies of lipoprotein nanoparticles (LPNs)

Targeting site	Targeting strategy	Targeting modification	Receptors	Cancer types	Key functions	References
Cancer cells targeting	Native receptor-mediated tumor targeting	LDL or HDL	LDLR, SR-BI	Multiple cancers, including breast, lung, prostate, cervical, hepatoma, etc.	Natural targeting based on the overexpression of LDLR and SR-B1 on cancer cells	[20, 43, 77– ﻿^81^], [15, 24, 35, 50, 53]
	Enhanced tumor targeting	BSA	GP60, SPARC receptors	Hepatoma, breast cancer	Alternative to apolipoproteins that provides natural targeting	[16, 45, 55]
		Folic acid-modified BSA	Folate receptors, GP60, and SPARC receptors	Various cancers	1.41-fold higher uptake compared to BSA alone	[45]
		Hyaluronic acid (HA)	CD44 receptor	Various cancers	No better targeting was achieved compared to uncoated nanoparticles	[26]
		Chondroitin sulfate (CS) coating	CD44 receptor	Various cancers	No better targeting was achieved compared to uncoated nanoparticles. Better than HA [50] due to its clathrin-mediated endocytosis-inducing ability	[26]
		CD47 antibody	CD47	TNBC	Competitively inhibits the binding of CD47 to the SIRPα and reverses immune escape.	[36]
		SDF1-mimic peptides (FH27, FH29, FH38)	CXCR4	Glioma tumor	Specifically target GICs and glioma tumors.	[30]
Cancer stem cells targeting	Native receptor-mediated tumor targeting	LDL or HDL	LRP-1	Cervical cancer stem cells, cervical cancer cells	Dual targeting of both cancer cells and cancer stem cells	[51]
TAMs targeting	Native receptor-mediated tumor targeting	HDL	SR-B1 on Mono-M2	Colorectal Cancer	Higher expression of SR-B1 receptors on TAMs	[39, 47]
		HDL	SR-B1 on M2-like TAMs/	TNBC	Higher expression of SR-B1 receptors on TAMs	[36]
	Enhanced tumor targeting	Legumain-sensitive melittin conjugate	legumain	Various cancers	Legumain enzyme is activated; Targeting the extracellular milieu, including TAMs.	[39, 43]
		Mannose	mannose receptor	breast cancer, lung cancer, prostate cancer	Specific targeting TAMs	[25]
		M2pep	Receptors on M2-like TAMs	TNBC		[42]
Stroma cells	Enhanced tumor targeting	Legumain-sensitive melittin conjugate	legumain	Various cancers	Legumain enzyme is activated; Targeting the extracellular milieu, including TAMs.	[39, 43]
		HC8 peptide		breast cancer, pancreatic cancer, colon cancer	Targeting different stroma cells (including ECs, CAFs, TAMs) simultaneously	[46]
		TNC and FN1-binding peptide	TNC, FN1	TNBC		[32]
Tumor vessels	Enhanced targeting	tLyP-1 peptide	NRP-1 receptor	TNBC	Promotes extravasation and deeper tissue penetration	[34, 40]
		CRGDfK peptide	αvβ3/5 integrins	Breast cancer cells	Enhanced extravasation from tumor vasculature	[41]
		RGD peptide	Integrins	Colorectal cancer, melanoma	Targeting neo-vasculature and enhancing cellular uptake	[10]
Lymphatic endothelial cells (LECs)	Enhanced targeting	PDPN targeted antibody	PDPN	various lymphatic endothelial cells, melanoma	Specifically target LECs.	[38]

### Facilitating transport across the blood-brain barrier (BBB)

The BBB is a protective shield formed by tightly joined brain endothelial cells that strictly regulates the entry of substances into the brain through tight junctions, efflux pumps (e.g., P-gp), and limited transcytosis, thereby blocking the entry of most drugs, especially large or hydrophilic molecules. LPNs can enhance drug delivery by disguising drugs, improving lipid solubility, or targeting BBB-specific transporters via surface modification, thereby promoting transcellular transport or bypassing efflux mechanisms to boost brain drug uptake [82]. Glioblastoma multiforme (GBM) is the most common and aggressive intracranial malignancy in adults. Radiotherapy is one of the current standard care for GBM patients. However, DNA repair and the limited distribution of reactive radicals in tumors contribute significantly to radiotherapy resistance. Furthermore, due to the BBB, most chemotherapeutics or other drugs cannot reach GBM tumor sites. GBM cells exhibit dysregulated cholesterol metabolism, with SR-B1 upregulated compared to normal brain cells, such as astrocytes. This, combined with LPNs’ small size (30–60 nm), which allows them to navigate biological barriers, makes it an ideal drug delivery vehicle for glioblastoma [33, 52]. Kapic et al. [52] designed a reconstituted HDL mimetic nanocomplex loaded with α-mangostin (AMN) for GBM treatment, defined as rHDL-AMN, which achieved IC50 values of 34.23 ± 6.5 µM (rHDL-AMN) and 38.25 ± 4.2 µM (free AMN), respectively, in the 3D GBM tumor model. In 3D normal astrocyte models, IC50 of rHDL-AMN was 60.72 ± 1.1 µM, while free AMN was 30.84 ± 2.5 µM, and the uptake in normal astrocytes with rHDL formulation was only < 3%, but 93% with free drug. AMN can also increase ROS, of which an overabundance and disruption of antioxidant homeostasis can promote cell death. In this study, free AMN showed a significant dose-dependent increase in ROS in normal astrocytes, while rHDL-AMN showed only marginal changes in ROS levels in astrocytes, indicating that rHDL-AMN protected normal cells from excessive autophagy and ROS generation. Sun et al. [33] added a C-C motif chemokine ligand 5 (CCL5) peptide on the surface of the bioinspired LPNs to further improve the ability to target GBM cells. CCL5 is highly expressed on the surface of GBM cells. Furthermore, they loaded reduction-sensitive NO donor and gemcitabine conjugate simultaneously, called C-LNG. During radiation therapy, NO donor molecules react with O2•− to form highly reactive cytotoxic ONOO− radicals, which have longer lifetimes, greater diffusion distances, and better spatiotemporal distributions compared with common ROS, thereby further sensitizing radiotherapy. The results showed that C-LNG demonstrated 2.45-fold higher fluorescence signals in brain tissues than the non-modified group. In vivo data showed that tumor volumes in the CCL5-Modified group were only 12.86% of those in the non-modified group at day 30. Zhang et al. [54] designed an HDL drug delivery system to load temozolomide (TMZ) and siRNA targeting pyruvate kinase M2 (siPKM2) for the treatment of GBM. Aerobic glycolysis is the main energy source of GBM. siPKM2 can reduce PKM2 protein levels, thereby inhibiting aerobic glycolysis in GBM and disrupting tumor cell energy supply. The histologic detection of glioma from hormonal nude mice after different treatments showed an obvious reduction of lactate and ATP in tumor sites.

Based on the significant selective targeting ability achieved by LPNs for GBM, researchers have expanded its application to other challenging brain cancers, particularly those affecting pediatric populations, where treatment considerations must balance efficacy with developmental concerns. Sonic hedgehog medulloblastoma (SHH MB) is a challenging type of childhood brain cancer. Standard treatment also uses brain radiation therapy, but it will lead to side effects on the normally developing brains of children. To address this, Kim et al. [44] designed engineered HDL-mimetic nanoparticles (eHNPs) loaded with LDE225 (a SHH inhibitor drug) and modified with anti-CD15 (stage-specific embryonic antigen-1, SSEA-1) to target brain SHH MB cells. The consistently stable formulation of multicomponent eHNPS-A1 and its derivatives, incorporating apoA1, anti-CD15, and LDE225, was achieved using microfluidic technology. The brain slice of the SHH MB mouse model treated with eHNP-A1 showed that the dual targeting of eHNP-A1 did enhance its ability to cross the BBB and reach MB cells.

### Drug release achieved by pH-responsive

**Fig. 4:**
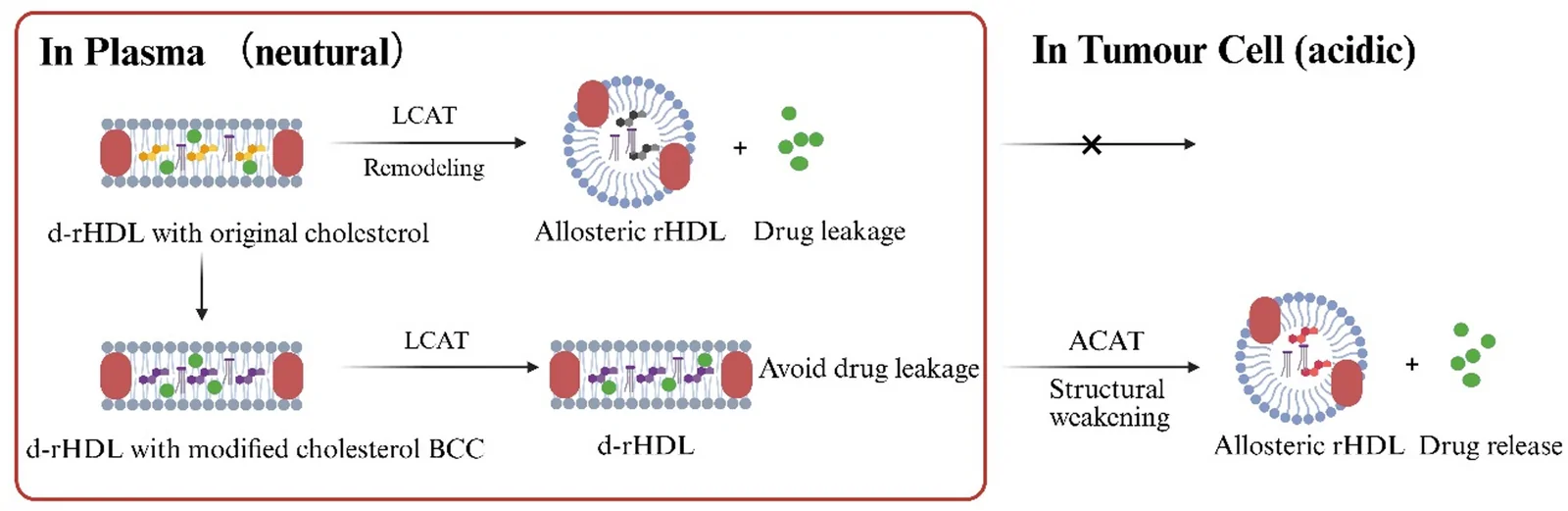
Substitute the original cholesterol in HDL discs (d-rHDL) with a modified cholesterol BCC. The original cholesterol undergoes an allosteric reaction catalyzed by serum LCAT, causing drug leakage. The acetal bond in BCC is pH-sensitive, thereby protecting BCC from LCAT. The acetal bond can be hydrolyzed in a tumor acid environment, converting BCC to free cholesterol. Then, free cholesterol undergoes esterification, catalyzed by ACAT in tumors, leading to the structural disintegration of d-rHDL and triggering precise intracellular drug release [16]

Physiological differences between normal tissue and tumor environments are significant. Normal tissue or blood environment is around neutral (pH = 7.4), while the pH of TME in malignant tumors is much more acidic (pH = 6.5–6.9), and the late endo/lysosomes have much lower pH, in the range 4.5–5.5 [83, 84]. Many drug delivery systems have been designed based on this mechanism. Duan et al. [27] designed a novel pH-responsive and sustained-release lipoprotein-like drug delivery system, BSA@CDs-DOX, loaded with doxorubicin (DOX). Carbon Quantum Dots (CDs), with a small size (2.5 nm), low toxicity, and fluorescent tracking function, serve as the primary carrier for DOX. CDs are rich in carboxyl groups (-COO-) that can bind DOX through electrostatic interactions and bind BSA through hydrophobic bonds. These bonds are relatively stable at pH 7.4 but can be cleaved at pH 5.0. The result shows that the cumulative release ratio of BSA@CDs-DOX at pH 5.0 was 7.5 times higher than at pH 7.4 in PBS solution. Li et al. [16] substituted the original cholesterol in HDL discs with a modified cholesterol BCC. The acetal bond in BCC is pH-sensitive and can be hydrolyzed, converting BCC to free cholesterol under the acidic conditions of TME. Then, free cholesterol undergoes an esterification reaction with linoleic acid, catalyzed by ACAT in tumors, leading to the structural disintegration of HDL discs and triggering precise intracellular drug release (Fig. 4).

**Fig. 5:**
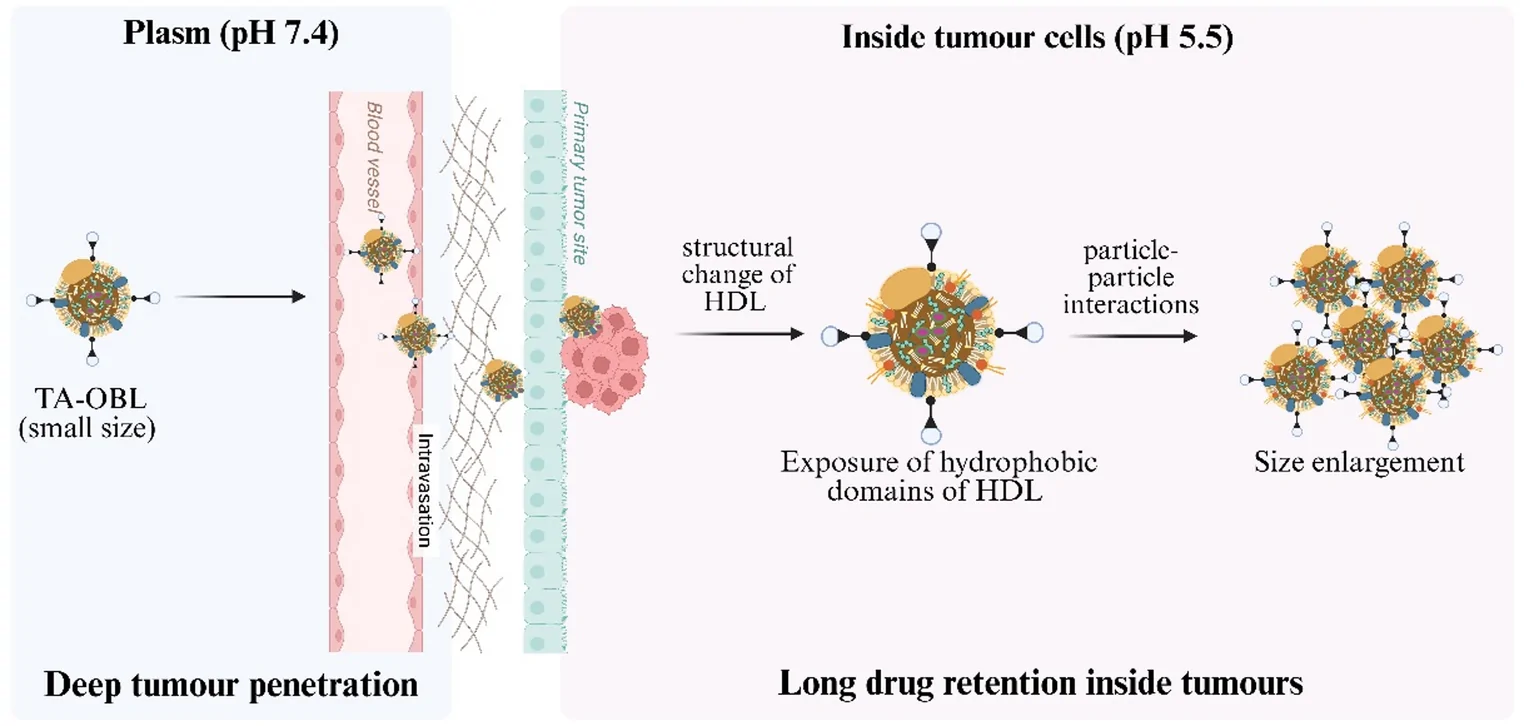
TA-OBL is designed to change size in response to pH by incorporating a pH-sensitive phospholipid PCP. TA-OBL remains small (~ 22 nm) at normal physiological pH (7.4) for deep tumor penetration but enlarges to over 300 nm inside the tumor cells for enhanced drug retention. This size transformation occurs because the PCP destabilizes in acidic conditions, exposing hydrophobic regions that promote particle aggregation to form much larger structures [43]

Additionally, pH-sensitive LPNs can achieve deep tumor penetration and prolonged drug retention simultaneously [42, 43]. By incorporating a pH-sensitive phospholipid PCP (1-palmitoyl-2-cycloglutaryl phosphatidylcholine), Li et al. [43] designed a size-enlargeable bioinspired LPNs (TA-OBL) to deliver oxaliplatin to cancer cells (Fig. 5). TA-OBL maintains a stable size of ~ 22.05 nm at normal physiological pH (pH 7.4), but when being exposed to tumor acidic conditions (pH 5.5), the initial size increased to ~ 226.3 ± 3.1 nm within 3 h and would further enlarge to over 300 nm after 24 h. The size change mechanism is that the cycloglutaryl group of PCP undergoes a structural change in acidic environments, leading to destabilization and exposure of the nanoparticles’ hydrophobic domains, which promote particle-particle interactions, causing the nanoparticles to aggregate and form much larger structures. In drug delivery systems, deep tumor penetration typically requires a small nanoparticle size, whereas prolonged drug retention and slow clearance demand relatively larger particles. CDs-DOX can satisfy both requirements simultaneously. The in vivo extravasation analysis of TA-OBL showed a largely co-localized signal with cancer cell regions, whereas the control group without PCP showed a signal that just halted at 20–40 μm from the cancer cell regions. The drug concentration in tumors of TA-OBL is 2.16-3.86-fold higher than that of the control group and can achieve an extended retention time of 60 h post-injection, when the control group has a negligible signal already. Tang et al. [42] used this theory in reverse. They achieved a particle size drop from 185.6 nm at pH 7.6 to 145.6 nm at pH 6.4 because of the acidic release of DOX@P particles in the nanoparticles, which allows for a long circulation time (achieved by larger initial size) and a deep tumor penetration up to 60 μm (achieved by smaller size).

### Monitoring distribution of nanoparticles/drugs in tumors

Monitoring the distribution of therapeutic agents is essential for developing more effective and personalized cancer treatments. This monitoring confirms whether therapeutics reach tumor sites in sufficient concentrations and enables adaptive dosing based on individual patient responses. Combining with imaging techniques such as PET and MRI, co-delivering therapeutic and diagnostic capabilities through LPNs is making the non-invasive, real-time, theranostic visualization of drug delivery a promising reality [28, 37]. Zheng et al. [37] designed a radiolabeled ^89^Zr-HDL allowing PET/CT imaging to track the distribution and tumor uptake of ^89^Zr-labeled HDL nanoparticles in nine esophageal cancer patients. The PET/CT imaging showed accumulation of ^89^Zr-HDL in esophageal tumors over time, with significantly higher uptake at 24 and 72 h compared to 1-hour post-injection. The effective radiation dose of ^89^Zr-HDL was 0.523 ± 0.040 mSv/MBq. The ^89^Zr-HDL PET/CT imaging technique could serve as a biomarker for predicting the efficacy of HDL-mediated drug delivery. This research combined ^89^Zr-HDL PET/CT with other imaging modalities (^18^F-FDG PET/CT, DWI/IVIM, and DCE-MRI) to provide a comprehensive assessment of tumor characteristics and nanoparticle uptake. They found the tumor uptake of ^89^Zr-HDL was not associated with ^18^F-FDG uptake, MRI measures of tumor characteristics, or quantitative measures of SR-B1 and CD68 expression in tumor biopsies, which indicated that the mechanism of HDL nanoparticle accumulation is not simply related to tumor metabolic activity and is also not primarily driven by tumor diffusivity, perfusion, or vascular leakiness, indicating a more complex mechanism than simple receptor-mediated uptake or macrophage engulfment. All these results may challenge the traditional view that nanoparticle accumulation in tumors is primarily due to the enhanced permeability and EPR effect. An alternative strategy to Zheng et al., Rakhshan et al. [28] used LDL nanoparticles to simultaneously deliver paclitaxel (PTX) and an MRI contrast agent (Gd-AAZTA-C17) to melanoma cells, enabling real-time visualization of nanoparticle uptake. Notably, the amount of internalized Gd in cell lines can be measured by inductively coupled plasma mass spectrometry (ICP-MS). Since the ratio of Gd to PTX in the nanoparticles is known, the PTX concentration can be estimated based on the measured Gd concentration.

## Improving immunotherapy with LPN drug delivery systems

Cancer immunotherapy has emerged as a promising treatment following traditional chemotherapy and radiotherapy. It marks a fundamentally different approach to treating cancer — by targeting the immune system rather than the tumor itself [85]. Cancer immunotherapy mainly includes immune checkpoint inhibitor (ICI) therapy, adoptive cellular immunotherapy, and tumor vaccines. However, ICIs are effective in only a limited subset of cancer patients, typically 10% to 40% [86, 87], indicating that immunotherapy still faces several critical challenges limiting its efficacy. A major obstacle is the robust tumor immunosuppressive microenvironment (TIME), which contains various immunosuppressive cells like myeloid-derived suppressor cells (MDSCs), regulatory T cells (Tregs), and M2-like TAMs [88]. These cells severely hamper the function of cytotoxic lymphocytes (CTLs) and contribute to tumor immune evasion. Additionally, the heterogeneous distribution of immune cells and dense stromal barriers in tumors restricts the delivery and penetration of immunotherapeutic agents [39, 88]. LPNs can be an effective strategy for addressing these issues. Efficient encapsulation of both hydrophilic and hydrophobic therapeutic agents enables the co-delivery of multiple immunomodulatory drugs with excellent biocompatibility, biodegradability, natural tumor-specific accumulation, and superior tumor penetration, thereby allowing LPNs to access deeper tumor regions and interact with various target sites to modulate TME [39, 88].

### Relieving immunosuppression and modulating microenvironment

#### Eliminating immunosuppressive cells directly

Some immunosuppressive cells, such as M2-like TAMs, highly express SR-B1. Therefore, drugs that kill M2-like TAMs can be selectively delivered via LPNs, thereby relieving the immunosuppressive environment and enhancing the efficacy of tumor immunotherapy. Monocyte-derived alternatively activated macrophages (Mono-M) are precursors to TAMs. As previously noted, M2-like TAM is tumor-promoting and dominant in TME, while M1-like TAM is antitumoral. Mono-M2 is the major source of M2-like TAMs. Mono-M2 can be recruited and differentiated at tumor sites, thereby inducing programmed cell death of tumor antigen-specific CD8 + T cells via the FasL-Fas pathway, creating a suppressive tumor immune microenvironment [25, 36, 42]. Due to the higher expression of SR-B1 receptors on Mono-M2 rather than other cells (such as Mono-M1) in the liver, Xiong et al. [47] developed a gemcitabine-loaded synthetic HDL (G-sHDL) that can preferentially target Mono-M2 to eliminate them and reverse immunosuppression in a mouse bearing colorectal cancer liver metastasis. Gemcitabine is an immunogenic cell death (ICD) inducer and thus promotes dendritic cell (DC) maturation. According to the co-culture of DCs and G-sHDL-treated tumor cells, G-sHDL can promote DCs’ maturation to enhance antitumor immunity. The combination of G-sHDL with an anti-PD-L1 antibody prolonged the survival of animals by 16 days compared with anti-PD-L1 alone (40 days vs. 24 days) without a significant effect on the mice’s body weights. Wang et al. [39] achieved a reduction of M2-like TAMs by designing a legumain-activating melittin-modified bioinspired nanovehicle. As mentioned above, active melittin is released when the nanovehicle encounters legumain in TME and thus increases the cellular uptake of the nanovehicle. This activation also upregulates NK cells and reduces M2-like TAMs. In addition, the nanovehicle incorporates theranostic photoradiotherapy (HPPH) and a ROS-responsive gemcitabine prodrug (RG). In the TAM, HPPH can act as a theranostic probe for in vivo tumor-targeting tracking. When nanovehicles are exposed to 671 nm laser light, HPPH generates ROS, leading to direct cancer cell death through oxidative damage, induction of immunogenic cell death (ICD), activation of DCs, and stimulation of anti-tumor immune responses. RG is designed with a ROS-sensitive thioketal linker that cleaves in the presence of ROS to release active gemcitabine, directly killing cancer cells and modulating the immune microenvironment by eliminating immunosuppressive cells, including MDSCs and M2-like TAMs. The in vivo studies verified that nanovehicles can increase T cell infiltration, modulate the immune microenvironment, reduce M2-like TAMs by 84.1% and MDSCs by 34.6%, and decrease the Treg population by 67.8%.

#### Reprogramming from M2-like macrophages to M1-like macrophages

The fate of macrophages depends on different environmental factors that drive them to become either pro-inflammatory M1-like macrophages or anti-inflammatory M2-like macrophages. Therefore, by regulating relevant factors, the mutual transformation of M2 and M1-like TAMs can be achieved. The process of macrophage polarization into either the M1 or M2 state is tightly controlled through multiple mechanisms, including signaling cascades, gene regulation, epigenetic modifications, and post-transcriptional control networks. Table 4 lists the factors that influence the polarization and reprogramming of macrophages [89]. M1-like TAMs can secrete pro-inflammatory cytokines, such as TNF-α, IL-6, and IL-1β, which function as antigen-presenting cells (APCs), facilitating the infiltration of cytotoxic T cells into tumors and enhancing the anticancer effects of clinical therapies. This is also associated with a favorable survival prognosis [47, 60, 66], so, in addition to directly reducing M2-like TAMs, reprogramming them to M1-like TAMs could also be an attractive way to modulate the TME. Dossou et al. [25] designed a TAM-targeting rHDL (rHDL-DPM-DMXAA) by coating the LPNs with mannose and loading them with an immunomodulatory drug, 5,6-dimethylxanthenone-4-acetic acid (DMXAA). Mannose receptor is highly expressed on the surface of TAMs, while DMXAA, also known as vadimezan, functions as a stimulator of interferon genes (STING) agonist contributing to the upregulation of IFN-β, CXCL10, and TNF-α, thereby promoting M1-like macrophage phenotype and enhancing DC maturation [50, 90]. After treatment with rHDL-DPM-DMXAA, in vitro results showed that M1 markers (IFN-β, CXCL10, TNF-α) increased and M2 markers (IL-10) decreased. Unfortunately, it was shown that DMXAA only binds to and activates mouse STING, not human STING. Zheng et al. [50] also encapsulated vadimezan and lenvatinib into synthetic HDL (LV-sHDL) for combinational immunochemotherapy against metastatic TNBC. Lenvatinib is a multitargeted tyrosine kinase inhibitor (TKI) that can induce ICD, inhibit immunosuppressive cells, prevent CD8 + T-cell exhaustion, and potentially downregulate PD-L1 expression on cancer cells through FGFR4 inhibition. Flow cytometry analysis revealed that LV-sHDL increased the M1-to-M2 macrophage ratio and enhanced intratumoral infiltration of total and active cytotoxic T lymphocytes by 33-fold and 13-fold, respectively, in the TNBC mouse model. Combining LV-sHDL with an anti-PD-L1 antibody further improved anti-tumor efficacy and extended median survival time to 38 days (compared to 24 days of anti-PD-L1 antibody alone). Dong et al. [36] designed a bionic LPN (BLP) loaded with chloroquine (CQ) and CD47 antibody (aCD47), named BLP-CQ-aCD47. Due to the high expression of SR-BI on M2 macrophages, BLP-CQ-aCD47 can be preferentially taken by M2-like TAMs. CQ acts as a reprogramming agent, which can polarize M2-like TAMs into M1-like TAMs. aCD47 functions as a targeting peptide to tumor cells and blocks the “do not eat me” signal produced by the combination of CD47 on the tumors’ surface and signal regulatory protein α (SIRPα) on the macrophage’s membrane, thus reversing the immune escape of tumor cells. The effects of macrophage polarization were assessed by increased expression of M1 markers (CD80, IL-6, IL-12, IFN-γ, TNF-α) and decreased expression of M2 markers (CD206, IL-10). Tang et al. [42] used imiquimod (R837), a toll-like receptor 7/8 (TLR7/8) agonist [53], as a reprogramming agent to repolarize M2-like TAMs into M1-like TAMs. They also modified the rHDL with an M2 macrophage-targeting peptide, M2pep, to improve targeting. Quantitative flow cytometry analysis showed that the M2/M1 macrophage ratio decreased from 2.83 to 0.52 compared with other control groups.

**Table 4:** Factors influencing the polarization and reprogramming of macrophages [89]

Factors	M1-like macrophages	M2-like macrophages
Stimulation	LPS, IFN-γ, GM-CSF, TNFα	LPS, ICs, GCs, LIF, adenosine, TGF-β, IFN-γ, IL-4, IL-10, IL-13, IL-33, Th cytokine, M-CSF, helminth
Cytokines	High TNFα, IL-1β, IL-6, IL-12, IL-18, IL-23”	Low IL-12, IL-23, high IL-1RA, IL-10, IL-4/IL-13
Markers	CD86, CD80, CD68, MHC II, IL-1R, TLR2, TLR4, iNOS, SOCS3	CD86, CD163, MHC II, SR, MMR/CD206, CD200R, TLR1, TLR8, VEGF, MMP9, TGM2, DecoyR, IL-1R, IIMR, Fizz-1, Ym1
Chemokines	CXCL1, CXCL3, CXCL5, CXCL8, CXCL9, CXCL10, CXCL11, CXCL13, CXCL16 CX3CL1, CX3CR1; CCL2, CCL3, CCL4, CCL5, CCL8, CCL11, CCL15, CCL19, CCL20; NOS2, CD64, IDO, SOCS1	CCL1, CCL2, CCL5, CxCL10, CCL13, CCL14, CCL17, CCL18, CCL22, CCL23, CCL24, CCL26, CXCL16, CCR2, CCR3, CCR4
Reprogramming	Reprogramming from M2 to M1; Superparamagnetic iron-oxide nanoparticles (SPIO), Glycocalyx-mimicking NPs (glycol-NPs), Ag, Au, Co, ZnO, TiO_2_, SiO_2_	Reprogramming from M1 to M2; hyaluronic acid-poly (ethylenimine), plasmid DNA NPs (HA-PEI/pDNA), miR-223-expressing plasmid DNA-encapsulated, HA-PEI NPs (HA-PEI/miR-223 NPs), alginate NPs, Au, Ti
Other macrophage-induced products	STAT1, STAT2, IRF5, p65, iron uptake, and metabolism; high ROS, ROIs, NO, and MHC I, low IL-10	STAT3, STAT6, IRF4, p50, iron release, folate uptake and metabolism, arginase 1 to polyamine

#### Other strategies to relieve immunosuppressive microenvironment

The anti-tumor immune response is primarily carried out by the adaptive immune response mediated by T cells. APCs capture antigens, migrate to lymphoid organs, and present tumor antigens to T cells, activating them into effector T cells. Tumor-specific effector T cells then migrate to tumor tissues, infiltrate, recognize, and ultimately kill tumor cells. ICIs promote the priming and activation of T cells and maintain their tumor-killing activity after T cells bind to tumor cells [86, 87]. Therefore, the prerequisite for ICIs to exert anti-tumor effects is the infiltration of T cells into tumor tissue, followed by binding and recognition of tumor cells [88, 91]. However, tumors develop sophisticated physical barriers that significantly impede CTL infiltration, thereby limiting therapeutic outcomes. Two major barriers are particularly noteworthy: the abnormal tumor vasculature and the dense stromal barrier. Wang et al. [46] developed stroma-cell-targeting bioinspired LPNs loaded with furoxans-oxaliplatin called S-LFO to overcome physical barriers in tumors. Stroma cell targeting was achieved by the HTTIPKVC (HC8) peptide modification. They also incorporated a reduction-sensitive NO donor conjugate by combining furoxans with oxaliplatin into HDL. It has been reported that a low concentration (< 100 nM) of NO in vascular endothelial cells (ECs) can promote tumor vessel normalization, thereby improving the effectiveness of anticancer therapies [46, 92]. Oxaliplatin can induce the ICD effect while NO released from S-LFO specifically reacts with reactive singlet oxygen (a type of ROS) to form ONOO-, which means consumption and inhibition of ROS, specifically its effects on the polarization of M2 macrophages [93]. The results also showed an 8.64-fold increase in the M1/M2 ratio compared to the PBS control group. In order to enhance drug delivery efficacy through tumor stroma and improve drug retention time in the TME, Wu et al. [32] designed a drug delivery system targeting increased fibronectin (FN1) and tenascin C (TNC) in tumor stroma by conjugating a TNC and FN1-binding peptide (FTF) to a bioinspired LPN. The FTF-modified system achieved a strong binding affinity to TNC and FN1 and maintained a significantly prolonged drug retention time in the tumor site even after 72 h post-injection. This system was also loaded with cabazitaxel and a pomalidomide prodrug as an immunomodulator. Upon exposure to fibroblast activation protein (FAP) in the TME, these drugs were released into the tumor ECM. The loaded pomalidomide then enhanced the proliferation and function of CD8 + T cells, further potentiating antitumor immunity.

### Direct activation of the immune system

#### Delivering tumor vaccines

Traditional vaccines mainly elicit humoral immunity, while cancer vaccines need to activate cellular immunity through CD8 + CTLs, so the delivery system needs to effectively target lymph nodes and DCs for strong antigen-specific CTL responses. HDL can be actively transported into lymphatic vessels via SR-BI receptors on lymphatic endothelial cells, and its small size (5–12 nm) allows it to penetrate deep into tissues. Liu et al. [53] developed a novel cancer nanovaccine targeting lymph nodes, an HDL nanoparticle loaded with the cancer cell membrane (antigen) and immune adjuvant (imiquimod/R837). They isolated two different cancer cell membranes as antigens from B16F10 adherent cells (ACs) against common cancer cells and B16F10 tumor sphere cells (TCs, a type of cancer stem cell) against more challenging, treatment-resistant cancers. After injecting the nanovaccine and harvesting the mice’s lymph nodes, they found that the nanovaccine group showed 2.1-fold higher accumulation in lymph nodes than the control group without the ApoA1-mimetic 22 A peptide. The flow cytometry results showed that memory T cells in lymph nodes, CD8 + T cells, and DCs were activated effectively by these nanovaccines. When mice were given a combination therapy with nanovaccines and anti-PD1 antibody, it showed an augmented anti-tumor efficacy compared with monotherapy. This nanovaccine platform could potentially achieve personalized therapy by using distinct cell membranes with specific antigens.

#### Activating immunogenic cell death (ICD)

ICD is a specific form of cell death that triggers an effective immune response against dying cells. When cells undergo ICD, dead tumor cells release tumor-specific antigens and damage-associated molecular patterns (DAMPs), which serve as danger signals to the immune system and thereby activate it. The key DAMPs include calreticulin (CRT), ATP, and high-mobility group box 1 (HMGB1). This process essentially turns dying cancer cells into a personalized vaccine, as the released signals stimulate DCs to present tumor antigens to T cells, ultimately activating an anti-tumor immune response. ICD not only kills cancer cells directly but also stimulates the body’s immune system to recognize and attack cancer cells, potentially leading to long-term anti-tumor immunity and better treatment outcomes. Various types of therapeutic agents can trigger ICD in cancer treatment, including traditional chemotherapy (doxorubicin, oxaliplatin, and paclitaxel), nanodrugs, traditional Chinese medicine (TCM), gas plasma systems, and radiation therapy [94]. Li et al. [43] used bioinspired LPNs (TA-OBL) to deliver oxaliplatin to cancer cells to enhance the anti-tumor effect of immune checkpoint blockade (ICB) by inducing ICD. However, the ICD effect relies on DCs presenting tumor antigens to T cells. Although DAMPs can stimulate DC maturation, their effects are generally weaker and less complete [95]. Therefore, the ICD effect can be further enhanced by overcoming the immunosuppressive TME and further activating the immature DCs. Cytosine-phosphate-guanine oligodeoxynucleotide (CpG ODN) is a novel synthetic single-stranded DNA immune adjuvant for cancer immunotherapy and a Toll-like receptor 9 (TLR9) agonist. TLR9 is primarily expressed on immune cells such as DCs and macrophages, and it can promote DC maturation, cytokine production, and T-cell responses. Once a CpG ODN activates TLR9, it provides the critical costimulatory signal that promotes DC maturation, inflammatory cytokine production (IL-12, TNF-α, IL-6), enhances tumor antigen presentation to T cells, and reverses the immunosuppressive microenvironment, thus leading to a robust immune response. Zhang et al. [95] compared the percentage of DC maturation among free ICD-inducing drug DOX, free CpG, and CpG-DOX combination, and dHDL-encapsulated CpG-DOX, and found an increased trend from 22.40%, 43.92%, 51.94%, to 87.51%, respectively. While the native CpG ODN is hydrophilic and does not naturally associate with the lipid-based HDL, Zhang et al. conjugated CpG ODN to DSPE by a heterobifunctional crosslinker maleimidobenzoyl-N-hydroxysuccinimide ester (MBS) and intercalated doxorubicin (DOX) into the CG base pairs of the CpG oligonucleotides based on π-π interactions between DOX and the base pairs. These π-π interactions are stable at physiological pH (7.4) with a modest drug release at tumor extracellular pH (6.8) and rapid release at tumor acidic pH (5.0). However, as a discoidal high-density lipoprotein (d-HDL), the researchers observed extracellular structural collapse of d-HDL upon interaction with SR-BI receptors, leading to drug release around the outside of tumor cells. Different from Zhang, Scheetz et al. [15] modified CpG with cholesterol (CpG-cholesterol), acting as a lipid anchor, to enable the CpG to be incorporated into the lipid-based sHDL nanoparticles, and co-delivered it with chemotherapy agents DTX to achieve a significant improvement in colon adenocarcinoma treatment. While the authors did not report the DC maturation rate, they observed significantly improved survival outcomes in MC-38 tumor-bearing mice, achieving a median survival of 43 days with the DTX-sHDL-CpG combination, compared to 28 days (*p* < 0.0001) for DTX-sHDL alone, and 23 days (*p* < 0.0001) for free DTX.

## Deliver nucleic acid agents by LPNs

### Delivering small interfering RNAs (siRNAs)

siRNAs are small, double-stranded RNA molecules, typically 20–25 nucleotides in length. The natural function of siRNA is to silence specific genes by triggering the destruction of their corresponding mRNA. Nucleic acid therapeutics are a powerful class of drugs for controlling gene expression and protein synthesis, with applications ranging from cancer treatment to infectious diseases. Despite significant advances that have led to several FDA-approved therapies, the clinical translation of nucleic acid drugs remains challenging. A major limitation is their poor stability in serum, where nucleases can rapidly degrade unprotected nucleic acids within hours. The hydrophilic and polyanionic nature also decreases their uptake by cells [96]. Additionally, the majority of nucleic acids that successfully enter cells become trapped within endosomes rather than reaching their cytoplasmic targets, severely limiting their therapeutic efficacy further. To overcome these delivery barriers, higher doses are often required, which can lead to side effects like thrombocytopenia and increased off-target effects. These challenges have contributed to relatively low success rates in clinical trials, with hundreds of trials yielding only 15 FDA-approved nucleic acid therapies to date. The development of effective delivery systems has emerged as a critical strategy to address these limitations. Nanocarrier-based delivery systems can protect nucleic acids from degradation, improve their pharmacokinetic profiles, enable targeted delivery to specific tissues and cells, and facilitate endosomal escape. LPNs, especially HDL, have shown promise due to their biocompatibility, enhanced cellular uptake, and the unique endo/lysosomal trapping bypass, which shields the nucleic acid cargo. The small size also allows them to evade clearance processes and penetrate tissues more effectively than naked nucleic acids. By engineering delivery systems to overcome these biological barriers, researchers aim to unlock the full therapeutic potential of nucleic acid drugs [30, 35].

**Fig. 6:**
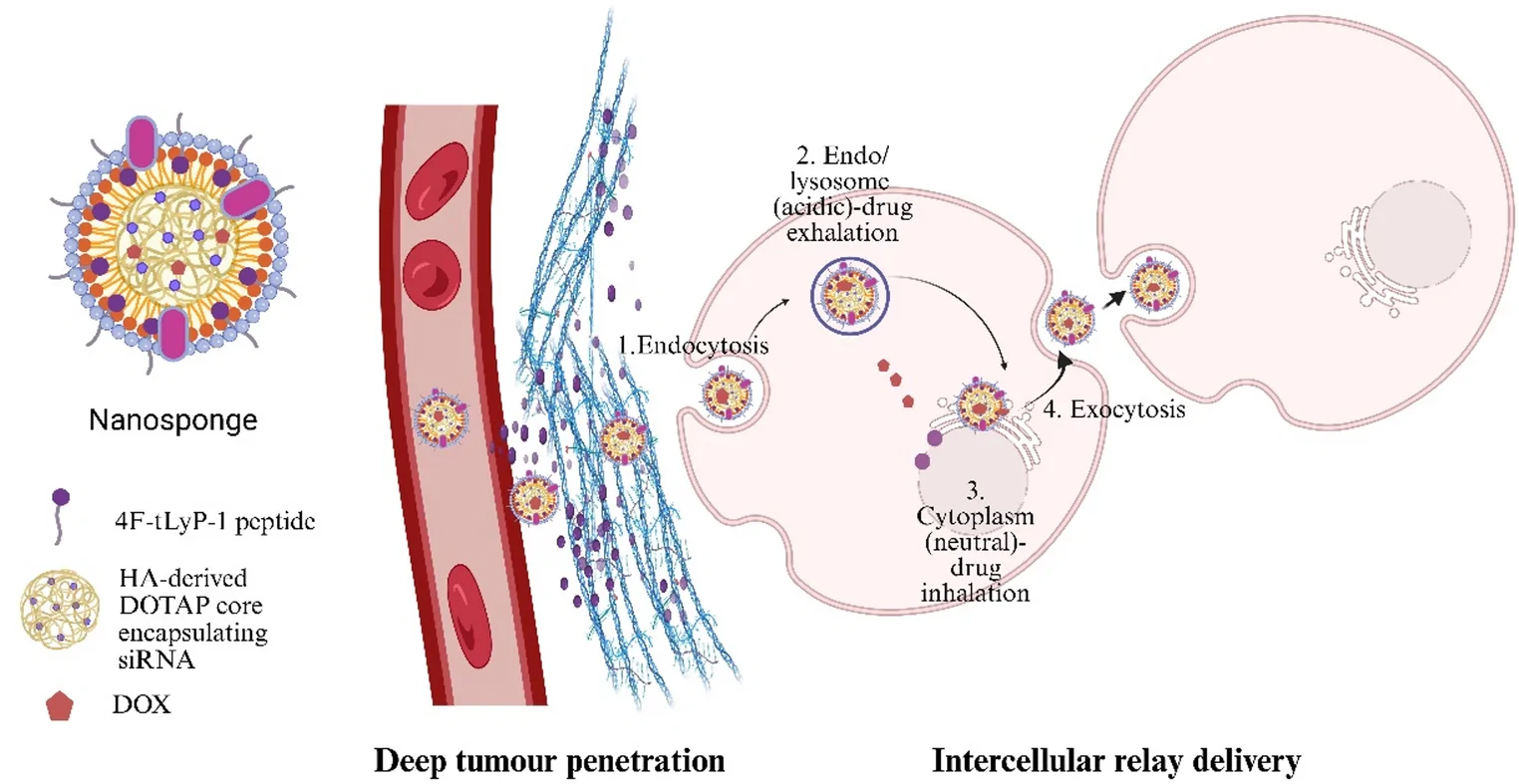
A hyaluronic acid (HA)-derived nanosponge allows intercellular-relay drug delivery. After being endocytosed into tumor cells, the nanosponge follows an endo-lysosomal pathway. The HA-derived polyampholyte core can undergo rapid protonation at lysosomal (pH 5.0), leading to dramatic swelling of the nanosponge. This swelling disrupts the lysosomal membrane, allowing the cargo to escape before significant degradation and thus protecting it. Then, the HA-derived polyampholyte core can reload the cargo at the cytoplasm (pH 7.4), be exocytosed via the Golgi apparatus to the extracellular space (pH 6.8), maintain DOX loading, and then be taken up by neighboring cells, allowing for a deeper tumor penetration, potentially reaching hypoxic areas that are typically resistant to treatment due to poor blood supply [34]

Yang et al. [34] designed an “enzyme-silenced nanosponge” to co-deliver siRNA and DOX. Consisting of a hyaluronic acid (HA)-derived polyampholyte core loaded with DOX, a cationic lipid layer encapsulating siRNA targeting hyaluronidase-1 (siHyal1), and a lipoprotein-inspired shell decorated with tumor-homing peptides. The cationic lipid DOTAP (1,2-dioleoyl-3-trimethylammonium-propane) is used to condense and encapsulate siRNA via electrostatic interactions. Silencing the enzyme Hyal1 helps protect the HA core of the nanosponge from degradation, thereby prolonging its lifespan. The phospholipid outer shell is decorated with 4 F-tLyP-1 fusion peptide, which combines two functional elements: 4 F (an ApoA-I mimetic peptide that further enhances the lipoprotein-like properties of the shell) and tLyP-1 (a tumor-penetrating peptide that targets the NRP-1 receptor overexpressed in tumor vessels). However, because the construct undergoes three-layer modification from core to shell, the fully assembled nanosponge, despite utilizing ApoA-Ⅰ to mimic HDL, has a size of around 130 nm, much larger than the original HDL (5–12 nm). In addition, the 4 F-tLyP-1 targeting peptide drives NRP-1-mediated uptake, which typically follows an endocytic pathway and may override or compete with the SR-BI selective uptake mechanism. Therefore, the author observed the nanosponge experiencing an endo/lysosomal route. However, the polyampholyte core can undergo rapid protonation at lysosomal pH (5.0), leading to dramatic swelling of the nanosponge. This swelling disrupts the lysosomal membrane, allowing the cargo to escape before significant degradation and thus protecting it. Additionally, this HA-derived polyampholyte core also allows an “intercellular relay drug delivery” of DOX (Fig. 6), which means the nanosponge can release loaded DOX at lysosome (pH 5.0), reload DOX at cytoplasm (pH 7.4), exocytosed via the Golgi apparatus to the extracellular space (pH 6.8), maintains DOX loading, and then be taken up by neighboring cells, allowing for a deeper tumor penetration, potentially reaching hypoxic areas that are typically resistant to treatment due to poor blood supply. With this HA polyampholyte core, the penetration improved from 29 μm (free DOX) to 59 μm.

Epithelial-mesenchymal transition (EMT) is the culprit of tumor invasion and metastasis. Snail is a significant transcription factor that plays a significant role in inducing EMT. Zhu et al. [24] designed a bioinspired LDL co-delivery system for doxorubicin (DOX) and siRNA targeting the Snail protein to achieve anti-metastatic effects. Based on the electrostatic interactions between negatively charged siRNA and calcium phosphate (CaP) in conjunction with the charge attraction between negative siRNA/CaP particles and positive liposomes (also containing DOTAP), they were able to incorporate siRNA into bio-LDL successfully. Western Blot analysis showed that this system effectively reduces Snail protein levels. In vitro cell migration (wound healing) assay results showed that the Apo-DOX-siRNA/CaP-Lips group exhibited significantly shorter migration distances compared with the group without siRNA. The cell invasion assay also confirmed that the Apo-DOX-siRNA/CaP-Lips group showed fewer invading cells. In vivo histological analysis also revealed fewer metastatic cells in liver and lung sections from the Apo-DOX-siRNA/CaP-Lips-treated group. However, this system may exhibit high drug release at physiological pH. Although the authors described it as a pH-responsive drug release system, in vitro studies showed that DOX was released at 55% at pH 7.4 and > 65% at pH 5.0 (24 h), while siRNA was released at 65% at pH 7.4 and 77% at pH 5.0 (24 h).

Nucleic acid drugs can also contribute to immunotherapy. Sakurai et al. [38] designed an LDL system to deliver PD-L1 siRNA (siPD-L1) to silence PD-L1 expression in lymphatic endothelial cells (LECs) and release the suppression of anti-tumor immunity of CTLs. The LECs’ targeting effect was primarily achieved by modifying LDL with a podoplanin (PDPN)- targeted antibody. PDPN is expressed specifically on the surface of LECs, and the targeting antibody was bonded by click reaction (azide-alkyne cycloaddition). Compared with the conventional amide coupling method, click reaction works in pH 4.0 and 4℃, where RNA is more stable, achieves higher conjugation efficacy (45.6% VS 9.6%), requires less antibody (0.75 equiv VS 3–10 equiv), reaches reaction plateau within shorter time (30 min VS 300 + minutes), and forms more stable triazole bonds resisting degradation in vivo. However, these drug delivery systems, including liposomes and non-covalent lipoprotein-based systems, typically achieve low nucleic acid densities of around 1–10 strands per particle due to their reliance on electrostatic binding, cholesterol-mediated binding, and other non-covalent interactions to incorporate DNA/RNA [35]. To address the low nucleic acid density issue, Sharma et al. [35] developed a lipid nanodisc based on covalent binding. In order to maximize the utilization of the nanodisc surface, they created binding sites on both the thiolated lipid surface and the cysteine-modified ApoA1 mimetic peptides perimeter of the nanodisc and effectively conjugated maleimide-modified antisense oligonucleotides (ASOs) that target hypoxia-inducible factor 1-α (HIF-1-α) mRNA to the binding sites through covalent thiol-maleimide chemical bonds instead of relying on weaker non-covalent interactions. Therefore, DNA was simultaneously conjugated to the top, bottom, and lateral belt of the nanodisc. The OliGreen assay results showed that the nanodiscs achieved a maximum of ~ 35 ± 14 DNA strands per particle on average, the highest reported density for such conjugates thus far.

### Delivering microRNA (miRNA)

While most studies have focused on the delivery of siRNA due to its highly specific characteristics, another small nucleotide miRNA can also act as a significant regulator of the expression and function of eukaryotic genomes. There are several key differences between siRNAs and miRNAs as therapeutic agents. siRNAs are typically designed for perfect complementarity with their target mRNAs, leading to direct cleavage and degradation of their target mRNA. miRNAs, on the other hand, usually exhibit imperfect complementarity to their targets and can regulate multiple genes simultaneously through translational repression and mRNA destabilization mechanisms. This broader targeting of miRNAs makes them potentially useful for diseases involving multiple dysregulated genes, but also increases the risk of unwanted effects. The choice between miRNA vs. siRNA approaches often depends on whether precise targeting of a single gene or broader regulation of a gene network is desired for the specific therapeutic application [97]. Jiang et al. [30] designed glioma-initiating cells (GICs) targeting LPNs to deliver miR34a. miR34a is physically entrapped within a calcium phosphate (CaP) core through electrostatic interactions between Ca²⁺ ions and the negatively charged miRNA. miR34a can simultaneously modulate multiple signaling pathways, such as suppressing cancer stemness, acting as a tumor suppressor in glioma, modulating the CD44 pathway, and reversing chemoresistance by modulating the Notch1 signaling pathway. The targeting effect was enhanced by binding SDF1-mimic peptides (FH27, FH29, FH38), which can interact with CXCR4, which is highly expressed on GICs and glioma tumor sites.

LPNs’ employment of distinct RNA incorporation strategies can impact RNA loading capacity and therapeutic efficacy. The electrostatic interaction between the negatively charged RNA agent and the positively charged part incorporated always achieves high colloidal and serum stability, with pH-responsive release of the RNA agent, resulting in substantial gene silencing. The positively charged mineral core entrapment can achieve a reproducible and quantifiable loading capacity of RNA with a smaller particle size. Jiang et al. reported miRNA loading of 1.21 ± 0.09% (w/w) with an encapsulation efficiency of 45.5 ± 3.5% with the particle size of 20–45 nm. Covalent conjugation strategies on LPNs, as demonstrated by Sharma et al., optimized oligonucleotide density and nuclease resistance. This approach achieved a significant increase in drug loading, approximately 35 ± 14 oligonucleotides per disc, which substantially exceeds the 1–10 strands per particle typically observed with noncovalent systems, ultimately achieving therapeutic efficacy at a single low dose.

## Conclusion and perspective

LPNs represent a significant advancement in addressing the persistent challenges of cancer therapy, such as poor solubility, limited targeting, and systemic toxicity. Their inherent biocompatibility, natural targeting capabilities, ease of modification, and versatility in accommodating diverse therapeutic agents position them as a transformative tool in the field of anti-tumor drug delivery. The evolution of LPN delivery strategies has progressed from simple drug encapsulation to multifunctional systems with precise targeting capabilities and stimuli-responsive behavior. The development of pH-sensitive, enzyme-responsive, intercellular relay drug delivery, and size-switchable LPNs demonstrates the field’s maturation, moving beyond merely mimicking natural structures toward engineering enhanced functionalities. Additionally, the diversity of therapeutic cargoes, ranging from conventional chemotherapeutics to immunomodulatory agents and nucleic acids, highlights the versatility and significant potential of LPNs.

Although the application of LPNs in delivering tumor treatment modalities has progressed significantly, evolving from proof-of-concept studies to sophisticated multifunctional nanoplatforms, the field still faces substantial challenges. Notably, despite decades of research and accumulated strong preclinical evidence, most LPN research in oncology remains at the threshold of first-in-human trials. While two HDL mimetics, CSL-112 and CER-001, have progressed to clinical trials, both are being developed for cardiovascular disease, sepsis, acute kidney injury, and rare lipid disorders [98, 99], rather than for cancer therapy. The primary obstacles to translational research include the complexity and high cost of large-scale production, with current methods such as thin-film hydration unsuitable for industrial manufacturing. Reproducibility represents a further concern. To date, most studies of LPNs in tumor treatment are highly individualized and lack independent validation by other research groups, while the in vitro and in vivo models employed in each study are typically limited in scope, thereby providing insufficient evidence that the results are applicable across a broader range of tumor models. Although most preclinical studies have demonstrated the biocompatibility and safety of LPNs, concerns regarding hepatotoxicity associated with their preferential hepatic accumulation persist, and extensive surface modifications are required to minimize these effects. Therefore, further research dedicated to enhancing the prospects for clinical translation is therefore essential and should be regarded as a priority direction for the field. For example, the development of scalable and reproducible manufacturing techniques, such as microfluidic assembly. Most encouragingly, however, the integration of imaging technologies and diagnostic capabilities with LPNs, enabling real-time monitoring of drug distribution, holds considerable promise and has progressed to be efficient among patients. The combination of diagnostic agents, therapeutic agents, and imaging technologies thereby enables non-invasive visualization of drug delivery, representing a significant step toward more precise and personalized cancer therapy and warranting further research in the field.

Furthermore, it is widely recognized that relying solely on SR-B1/LDLR-mediated targeting is far from satisfactory, as LPNs often exhibit liver-biased biodistribution. Many studies have reported several promising targeting peptides, but it is rarely mentioned that conjugating a targeting peptide, particularly through surface modification, often increases the risk of triggering immune reactions. Chemical conjugation methods may impair peptide function when targeting moieties are attached to carrier surfaces, potentially reducing targeting efficiency. Adding targeting ligands also uplifts manufacturing/quality-control burdens and potentially toxicity levels. Jumping out of the peptide-based targeting strategies, utilizing the natural tumor tropism of endogenous carriers/molecules is an alternative. Tang et al. [100] utilize the natural tumor tropism of mesenchymal stem cells (MSCs) and conjugate nanoparticles directly to MSCs via a thioether bond (the maleimide groups on the nanoparticles react with thiol groups on the MSCs’ membranes. The inherent tumor-homing capability of MSCs, driven by chemokine gradients and tumor-derived inflammatory signals, causes nanoparticles-conjugated MSCs to migrate toward tumor sites naturally. Zheng et al. [101] coated engineered macrophage membranes, which provide inflammation-directed chemotaxis, onto nanoparticles, enabling simultaneous targeting of both primary tumors and multiple metastatic lesions and significantly reducing systemic toxicity and immune clearance compared to some peptide-receptor-mediated tumor targeting.

Assisting cancer immunotherapy with LPNs has attracted significant attention in recent years and is demonstrating significant progress in overcoming the immunosuppressive TME. Current achievements include the successful reprogramming of TAMs from immunosuppressive M2 to anti-tumor M1 phenotypes, enhanced dendritic cell maturation and antigen presentation, effective delivery of cancer vaccines with improved lymph node targeting, and the induction of ICD effect leading to durable anti-tumor immunity. However, the complex and dynamic nature of immune responses still poses substantial challenges in realizing the full potential of these systems, such as the variable expression of immune cell markers on different immune-related cells, which affects targeting efficiency, and a limited understanding of optimal timing and sequencing for immunotherapy delivery. Looking forward, seeking effective immunomodulatory agents, combining chemotherapy and immunotherapy, introducing cancer vaccines or RNA therapy for synergistic effects, creating personalized platforms based on patient-specific tumor and immune profiles, and introducing artificial intelligence for predictive modeling and repeatable manufacturing standardization of drug delivery systems, positions LPN as a promising and transformative approach for next-generation cancer immunotherapy.

The co-delivery of nucleic acid agents with chemotherapeutics and/or immunotherapeutics through LPNs represents one of the most promising frontiers in cancer therapy. However, in addition to the inherent challenges posed by the delicate nature of nucleic acid agents, delivering them with LPNs also faces challenges, which restrict most applications to preclinical research. First of all, the loading method of the nucleic acid agent. The most commonly used method is through the electrostatic interactions. By precomplexing a cationic part with the negatively charged RNA molecules, then introducing the complex into LPNs. However, introducing such a complex poses a threat to the size of the nanoparticles, thereby affecting their penetration and drug delivery. Although Sharma et al. [35] reported loading RNA through a covalent bond, which is more stable and allows a high loading capacity, they had to compromise on the multi-step synthesis process, which may potentially damage the delicate RNA structure, affecting RNA releasing profile, and almost close the door for scalable clinical manufacturing. Delivering RNA therapeutics is a cutting-edge field and still requires more attention, but what attracts us more is that, compared with the temporary and even reversible treatment effect generated by siRNA/mRNA at the level of RNA/protein, which requires continuous administration, gene editing technologies such as CRISPR-Cas9 could generate an irreversible and one-time treatment at the level of DNA. However, there is a rare report researching CRISPR-Cas9 genome editing using LPNs for cancer therapy. The most commonly used vectors for CRISPR-Cas9 genome editing are viral vectors and lipid nanoparticles. However, both delivery systems face significant limitations that require further development [85–87]. Viral vectors present strong immunogenic responses and limited packaging capacity. Similarly, lipid nanoparticles exhibit reduced targeting ability and lower transfection efficiency. In contrast, LPNs offer several theoretical advantages that could position them as promising alternatives to gene-editing technologies, including favorable biocompatibility, improved cargo-loading capacity, and diverse targeting and modification potential. These characteristics suggest that LPNs may represent a viable and effective vector option for advancing CRISPR-Cas9 applications.

Although we have discussed the current research status of chemotherapy, immunotherapy, and gene therapy separately, we can tell that the most promising treatment is, no doubt, combination therapy involving two or more of these modalities, and such co-delivery systems can simultaneously address tumor treatment challenges like drug resistance and tumor immune suppression in ways that single-agent therapies cannot achieve.

In conclusion, although substantial progress has been achieved in the design and application of LPNs for cancer therapy, fully realizing their clinical potential will require overcoming key challenges. With continued research, these promising drug delivery platforms hold great potential for advancing more effective, targeted, and personalized cancer treatment strategies.

## Data Availability

No datasets were generated or analysed during the current study.
